# An Intelligent Track Segment Association Method Based on Characteristic-Aware Attention LSTM Network

**DOI:** 10.3390/s25113465

**Published:** 2025-05-30

**Authors:** Jiadi Qi, Xiaoke Lu, Jinping Sun

**Affiliations:** 1 Nanjing Research Institute of Electronic Technology, Nanjing 610500, China; qjd0809@126.com; 2 School of Electronic and Information Engineering, Beihang University, Beijing 100191, China; sunjinping@buaa.edu.cn

**Keywords:** sensor data processing, track segment association, characteristic-aware attention, gated adaptive long short-term memory

## Abstract

Accurate track segment association plays an important role in modern sensor data processing systems to ensure the temporal and spatial consistency of target information. Traditional methods face a series of challenges in association accuracy when handling complex scenarios involving short tracks or multi-target intersections. This study proposes an intelligent association method that includes a multi-dimensional track data preprocessing algorithm and the characteristic-aware attention long short-term memory (CA-LSTM) network. The algorithm can segment and temporally align track segments containing multi-dimensional characteristics. The CA-LSTM model is built to perform track segment association and has two basic parts. One part focuses on the target characteristic dimension and utilizes the separation and importance evaluation of physical characteristics to make association decisions. The other part focuses on the time dimension, matching the application scenarios of short, medium and long tracks by obtaining the temporal characteristics of different time spans. The method is verified on a multi-source track association dataset. Experimental results show that association accuracy rate is 85.19% for short-range track segments and 96.97% for long-range track segments. Compared with the typical traditional method LSTM, this method has a 9.89% improvement in accuracy on short tracks.

## 1. Introduction

In modern military defense and air traffic control systems, multiple networked radars and sensors are deployed to form a system that can achieve multi-dimensional data processing and target tracking. As a core technology in radar data processing, multi-sensor track segment association and fusion is the key to situational awareness [1], directly determining the system’s global perception accuracy and decision reliability. With the widespread application of distributed heterogeneous sensors, track data will be affected by error superposition and spatiotemporal asynchrony [2].

Traditional track segment association methods are categorized into probabilistic statistical, fuzzy mathematical, and anti-bias tracks association algorithms. Probabilistic statistical methods, such as hypothesis testing-based methods, evaluate track correlation through statistics. Probabilistic statistical track segment association algorithms, such as weighted methods and statistical double threshold approaches, show good performance under low noise conditions. Wang et al. [3] solved the sensor-target count mismatch problem by using sequential m-best algorithms with cost likelihood function refinements. However, the real-time performance of such methods degrades due to the combinatorial explosion in dense target environments [2,4]. Li et al. [5] optimized the distance weight allocation of the optimal sub-pattern assignment (OSPA) and introduced a sliding window function. Wang et al. [6] proposed an engineering-oriented probabilistic data association (JPDA) algorithm to improve the data association accuracy and practicality in multi-target tracking scenarios. Fuzzy mathematical methods, including grey relational analysis, enhance adaptability to complex environments [7]. Xiong et al. [8] constructed multidimensional grey relational matrices with a dynamic feedback mechanism to mitigate the impact of time-varying noise. Guo et al. [9] reduced computational complexity in asynchronous multi-node scenarios via a hybrid virtual-real sequence temporal divergence algorithm. Su et al. [10] achieved track clustering using fuzzy membership functions. Chen et al. [11] proposed an asynchronous track-to-track association method based on pseudo-nearest neighbor distance and grey correlation theory. Zhang et al. [12] proposed to take the advantages of communicable collaboration in a peer-to-peer structure and establish an error correction model between high-precision and low-precision nodes. However, fuzzy mathematics methods are dependent on parameter settings and cannot adapt to dynamically changing complex environments. Anti-bias tracks association algorithms focus on reducing sensor biases and false alarms. Yang et al. [13] designed visual equivalence factors based on target measurement error distributions for cross radar associations using classical assignment methods. Cui et al. [14] developed generalized spatiotemporal intersection matching models to suppress regional bias effects. Zhang et al. [15] enhanced association stability in dense clutter environments through relative coordinate allocation matrices (RCAM). However, the robust association method is not capable of modeling features and cannot be generalized.

Breakthroughs in the field of deep learning have provided new methods for track segment association and fusion [16]. With their powerful characteristic extraction and nonlinear modeling capabilities, these methods achieve data-driven association decisions [17,18]. Deep learning architecture substantially overcomes the limitations of traditional approaches caused by dependence on specific motion models, allowing it to avoid the impact caused by the difference between the hypothetical model and the complex track scenarios in the real world. Convolutional neural networks (CNNs) and long short-term memory (LSTM) networks effectively exploit dynamic temporal patterns in tracks and reduce the reliance of traditional methods on predefined motion models [19,20]. Generative adversarial networks (GANs) and contrastive learning further enhance the robustness of discontinuous track segment association [21,22]. However, current research predominantly focuses on single scenario associations, and areas such as end-to-end fusion frameworks and multi-scene adaptive algorithms need further exploration [23].

Multilayer perceptions (MLPs) were initially introduced for time series classification (TSC) tasks due to their simplicity, but they struggled to capture temporal dependencies and multi-scale characteristics [24]. Variants of CNNs, such as multi-channel deep convolutional neural networks (MC-DCNN), can handle multivariate data by processing each channel independently [25]. Fully convolutional network (FCN) enhances input flexibility through global average pooling [26]. Through the modular network architecture, multi-level models based on specific networks and parallel fusion structures are also proposed [27,28]. Cao et al. [29] proposes the contrastive Transformer encoder representation network (TSA-cTFER) based on contrastive learning and Transformer encoder, integrating a two-stage online algorithm for dynamic management of track segment association.

To address these challenges, this paper proposes an association data preprocessing algorithm and the CA-LSTM model. The model establishes a track segment association method through the collaborative optimization of multi-scale dilated convolution, characteristic-aware attention, and gated adaptive LSTM. The main contributions are as follows:

- This paper developed an association data preprocessing algorithm that can segment and time-series synchronize the data from different sensors within the same time window. At the same time, different tracks are combined to form inputs suitable for the model.
- The CA-LSTM model optimizes characteristic representation through three mechanisms: characteristic group embedding, encoding, and characteristic-aware attention. This method can assign characteristic weights based on model training results, effectively solving the waste of multi-dimensional data caused by using only position characteristic data in traditional methods.
- The model combines a gated adaptive LSTM and a multi-scale dilated convolution to optimize the representation of the time dimension. The two modules can extract the characteristics of the input in the time dimension, and obtain the short-range and medium-range correlations of adjacent nodes and the long-range correlations of the entire track.

Verification on the multi-source track segment association dataset shows that the proposed method outperforms existing deep learning models in core metrics. This method provides a technical method for intelligent collaborative sensing technology in distributed sensor networks.

## 2. Track Segment Association Problem Formulation

### 2.1. Problem Description

In multi-sensor information fusion systems, track segment association aims to correlate track data from heterogeneous sensors to the same physical target while eliminating systematic biases. Let source α, for example, radar station A, provide a track set $\Theta_{\alpha }=\left\{U_{1}^{\alpha },\cdots ,U_{i}^{\alpha },\cdots ,U_{n}^{\alpha }\right\}$ containing n tracks, and source β, for example, radar station B, provides $\Theta_{\beta }=\left\{U_{1}^{\beta },\cdots ,U_{i}^{\beta },\cdots ,U_{m}^{\beta }\right\}$ with m tracks. Each track comprises sequential state observations

(1) $$U_{i}^{s}=\left\{X_{i,1}^{s},X_{i,2}^{s},\cdots ,X_{i,k}^{s},\cdots |k\in K\right\},$$

where $s\in \left\{1,\cdots ,\alpha ,\cdots ,\beta ,\cdots \right\}\;$ denotes the sensor source, i is the track index, and K represents the temporal index set. $X_{i,k}^{s}=[t_{i,k}^{s},x_{i,k}^{s},y_{i,k}^{s},\cdots ,v_{i,k}^{s},\cdots ,\theta_{i,k}^{s},\cdots ]$ defines the state vector at time $t_{i,k}^{s}$, including Cartesian coordinates (x,y,z), velocity v, heading angle θ, and some other dimension characteristic data. Assume that the number of characteristics is represented by parameter τ, and the dimension of vector $X_{i,k}^{s}$ is 1×τ.

The core objective is to derive an optimal mapping function $\hat{\omega }:\Theta_{\alpha }\to \Theta_{\beta }\cup \{0\}$ which associates each track $U_{i}^{\alpha }$ from source α to its counterpart $U_{j}^{\beta }$ from source β, or labels it as unassociated. This reduces to a binary classification task for track pairs $\left(U_{i}^{\alpha },U_{j}^{\beta }\right)$ to determine their association likelihood.

### 2.2. Association Characteristic Processing Algorithm

In actual scenarios, significant heterogeneity exists in track data acquisition from different sensors. Some sensors in the network operate at higher refresh rates, while others have latency. Variations in update cycles can cause track points to be inconsistent in time and have differences in the number of observations within the same time interval. These differences require temporal alignment through data preprocessing methods.

The alignment process begins with the association data processing module, which filters and combines the raw association results. By analyzing the data characteristics, specific track data combinations meeting predefined criteria are selected, establishing a foundation for subsequent processing. In the time alignment part, the shared temporal coverage is first determined for different sensors’ data matrices. This involves identifying the minimum and maximum timestamps across matrices to define a common time interval. Data within this interval are then filtered and aligned using window-based operations. For example, given a row of characteristic data from sensor A with timestamp t, a time window centered at t is defined as the data of sensor B. The average value of the relevant characteristics within this window is calculated to achieve preliminary time alignment. Subsequently, the scenario data processing function groups data by source identifiers and batch numbers. Using the results from the associated data processing stage, corresponding grouped data undergo further alignment and merging operations. This ensures the consistency of the multi-sensor dataset in structure and time.

Through these preprocessing steps, challenges caused by sensor temporal inconsistencies and observation quantity differences are effectively mitigated. The implemented temporal alignment provides a unified and accurate data foundation for subsequent track-based analysis and applications.

The temporal data alignment and windowing process is initiated as follows, for two temporal data matrices A and B, where matrix A consists of the state vectors of the track $U_{i}^{\alpha }$ connected in time order, and matrix B is constructed in a way similar to track $U_{j}^{\beta }$. The shared temporal range $\left[t_{\mathrm{start}},t_{\mathrm{end}}\right]$ is first determined by formula

(2) $$t_{\mathrm{start}}=\max\left\{\underset{i=1}{min}\;t_{i,k}^{\alpha },\underset{i=1}{min}\;t_{j,l}^{\beta }\right\},\;t_{\mathrm{end}}=\min\left\{\underset{i=1}{max}\;t_{i,k}^{\alpha },\underset{i=1}{max}\;t_{j,l}^{\beta }\right\},$$

where $t_{i,k}^{\alpha }\in U_{i}^{\alpha },t_{j,l}^{\beta }\in U_{j}^{\beta }$. The data matrices $A_{\mathrm{range}}$ and $B_{\mathrm{range}}$ are filtered out within this time range, and a numerical column aggregation alignment operation is performed on the data matrices to finally obtain ${\tilde{B}}_{\mathrm{range}}$. The calculation is as follows

(3) $$A_{\mathrm{range}}=\left\{X_{i,k}^{\alpha }\in A|t_{i,\mathrm{start}}\leq t_{i,k}\leq t_{i,\mathrm{end}}\right\},$$

(4) $$B_{\mathrm{range}}=\left\{X_{j,l}^{\beta }\in B|t_{j,\mathrm{start}}\leq t_{j,l}\leq t_{j,\mathrm{end}}\right\},$$

(5) $${\tilde{X}}_{j,k}^{\beta }=\left\{\begin{array}{cc} \frac{1}{\left|B_{\mathrm{range}\;}\right|}\sum_{X_{j,l}^{\beta }\in B_{\mathrm{range}}}X_{j,l}^{\beta ,}, & \left|B_{\mathrm{range}\;}\right|>0\\ \;\emptyset , & \left|B_{\mathrm{range}\;}\right|=0 \end{array}\right.,$$

(6) $${\tilde{B}}_{\mathrm{range}\;}=\left\{{\tilde{X}}_{j,k}^{\beta }\in B|t_{j,\;\mathrm{start}\;}\leq t_{j,k}\leq t_{j,\;\mathrm{end}\;}\right\}.$$

At this time, the dimension of matrix $A_{\mathrm{range}}$ and matrix ${\tilde{B}}_{\mathrm{range}}$ is 1×τ·K. In the window division stage, the number of windows n is calculated based on predefined window size w and sliding step δ

(7) $$n=\left\lceil \frac{\left|K\left(A_{\mathrm{range}}\right)\right|-w}{\delta }\right\rceil +1,$$

where $K\left(A_{\mathrm{range}}\right)$ denotes the index count of track data matrix $A_{\mathrm{range}}$. In the subsequent experiments in this study, the window size is generally determined to be the length of the multidimensional data of the track segment. Assuming that the track segment is a 10-point track segment, the window size w is defined as w=10·τ. The sliding step size is set as $\delta =\frac{w}{5}$, one fifth of the window length. This ensures that the track segment data generates more training samples, enhances the robustness of the model, and does not increase the computational cost too much while covering data changes.

For each window, the start and end indices are computed as

(8) $$\;start=k\times \delta ,\;end=\min\left(\mathrm{start}+w,\left|K\left(A_{\mathrm{range}}\right)\right|\right).$$

After extracting windowed data, matrices $A_{\mathrm{range}}$ and ${\tilde{B}}_{\mathrm{range}\;}$ are combined in chronological order to form the windowed result matrix $D_{\mathrm{window}}$. This structured windowing process facilitates setting a standardized data format for subsequent analysis and model training.

Differential processing is applied to paired track data within $D_{\mathrm{window}}$ to compute column-wise differences

(9) $$\left\{\begin{array}{c} \Delta x_{\gamma ,k}=x_{\gamma ,k}^{\alpha }-x_{\gamma ,k}^{\beta }\\ \Delta y_{\gamma ,k}=y_{\gamma ,k}^{\alpha }-y_{\gamma ,k}^{\beta }\\ \cdots \\ \Delta v_{\gamma ,k}=v_{\gamma ,k}^{\alpha }-v_{\gamma ,k}^{\beta }\\ \cdots \\ \Delta \theta_{\gamma ,k}=\theta_{\gamma ,k}^{\alpha }-\theta_{\gamma ,k}^{\beta }\\ \cdots \end{array}\right.,$$

where γ denotes the γ-th window’s track data, γ∈1,2,⋯,n, k represents the k-th timestamp within the track segment, k∈1,2,⋯,w, and $x_{\gamma ,k}^{\alpha }$ indicates the longitude value at the k-th timestamp in the γ-th window of sensor α.

Normalization is subsequently executed. Taking longitude $x_{\gamma ,k}$ as an example following

(10) $${\hat{x}}_{\gamma ,k}\left\{\begin{array}{c} \frac{x_{\gamma ,k}-x_{\gamma }^{min}}{x_{\gamma }^{max}-x_{\gamma }^{min}},\;\;\;\;\;\;\;\;x_{\gamma }^{max}\neq x_{\gamma }^{min}\;\;\\ 0,\;\;\;\;\;\;\;\;\;\;\;\;\;\;\;\;\;\;\;\;\;\;\;\;x_{\gamma }^{max}\neq x_{\gamma }^{min}\; \end{array}\right.$$

$x_{\gamma }^{max}$ represents the maximum value in $x_{\gamma ,k}$, and $x_{\gamma }^{min}$ represents the minimum value in $x_{\gamma ,k}$. Latitude $y_{\gamma ,k}$, speed $v_{\gamma ,k}$, and heading $\theta_{\gamma ,k}$ undergo analogous normalization. These preprocessing steps produce standardized data suitable for subsequent analysis and modeling tasks. The algorithm adopts a sequential architecture for track segment association analysis, as detailed in Algorithm 1.
**Algorithm 1:** Association characteristic processing algorithm**Input**        : Sensor matrices A(α), B(β), window size w, slide step δ $\mathrm{Output}\;\;\;\;\;:\mathrm{Windowed}\;\mathrm{datasets}\;D_{\mathrm{window}}$       $D_{\mathrm{window}}$$\;=\;\emptyset ,\;{\tilde{B}}_{\mathrm{range}}$ = ∅       $t_{\mathrm{start}}$ = max (min ($t_{i,k}^{\alpha }$, min ($t_{j,l}^{\beta }$))       $t_{\mathrm{end}}$ = min (max ($t_{i,k}^{\alpha }$), max ($t_{j,l}^{\beta }$))       $A_{\mathrm{range}}$$\;=\;A\;[t_{\mathrm{start}}\;$$:\;t_{\mathrm{end}}$$],\;B_{\mathrm{range}}$$\;=\;B\;[t_{\mathrm{start}}$$\;:\;t_{\mathrm{end}}$]       n = ⌈((length of $A_{\mathrm{range}}$) − w)/δ⌉ + 1       **for** γ = 1: n **do**              s = (γ − 1) * δ, $e\;=\;\min\;(s\;+\;w,\;\mathrm{length}\;\mathrm{of}\;A_{\mathrm{range}}$)              $A_{\mathrm{window}}\;$$={\;A}_{\mathrm{range}}$$[s:e],\;B_{\mathrm{window}\;}$$=\;B_{\mathrm{range}\;}$[s:e]              **if** $B_{\mathrm{window}}\;$≠ ∅ **then**                    ${\tilde{B}}_{\mathrm{window}}$$\;=\;\mathrm{mean}(B_{\mathrm{window}}$)             **else**                    ${\tilde{B}}_{\mathrm{window}\;}$= ∅             **if** ${\tilde{B}}_{\mathrm{window}\;}$≠ ∅ **then**                    **for** f = x, y, v, θ **do**                            $\Delta f\;=\;A_{\mathrm{window}}[f]$$\;-\;{\tilde{B}}_{\mathrm{window}\;}[f]$                            $\hat{f}$ = (Δf − min(Δf))/(max(Δf) − min(Δf))                    **end**             **end**             $\mathrm{add}\;(A_{\mathrm{window}}$$,\;\hat{f}$$)\;\mathrm{to}\;D_{\mathrm{window}}$       **end**       $\mathrm{return}\;D_{\mathrm{window}}$



### 2.3. Association Decision Output

The association decision uses a specific algorithm to evaluate the track data in pairs to measure the correlation or other association metrics between the tracks. If a pair of tracks can be determined to belong to the same target according to predefined criteria and thresholds, they are labeled as positive class, usually represented by 1. Conversely, if the pair is considered to represent different targets, it is labeled as a negative class, which is typically denoted as 0. For a given track pair $\left(U_{i}^{\alpha },U_{j}^{\beta }\right)$, the association decision algorithm calculates an association metric value $M(U_{i}^{\alpha },U_{j}^{\beta })$. The boundary between association and non-association is divided by a pre-defined threshold θ.

The association decision output $L(U_{i}^{\alpha },U_{j}^{\beta })$ is formally defined as a binary classification label

(11) $$L\left(U_{i}^{\alpha },U_{j}^{\beta }\right)=\left\{\begin{array}{cc} 1, & M\left(U_{i}^{\alpha },U_{j}^{\beta }\right)\geq \theta \\ 0, & M\left(U_{i}^{\alpha },U_{j}^{\beta }\right)<\theta \end{array}\right.,$$

where $L(U_{i}^{\alpha },U_{j}^{\beta })$ denotes the output label for the track pair $\left(U_{i}^{\alpha },U_{j}^{\beta }\right)$, and θ is a threshold determined by empirical or theoretical analysis. The analysis is determined according to specific application scenarios and data characteristics.

In deep learning approaches, the track characteristics to be adjudicated are fed into a trained classifier that outputs binary labels. The input track data tensor x undergoes characteristic transformation and nonlinear mapping through fully connected layers and leaky rectified linear unit activation function $LeakyReLU\left(\cdot \right)$, thereby generating an output vector y

(12) $$y=W_{2}\;LeakyReLU\left(W_{1}x+b_{1}\right)+b_{2},$$

where $W_{1}$ denotes the first layer weight matrix and $W_{2}$ denotes the second layer weight matrix. $b_{1}$ and $b_{2}$ represent the bias vectors of the two layers, respectively. The output vector y is converted into a probability vector p through the soft maximum function $Softmax\left(\cdot \right)$, and the final classification label is determined by comparative probability values following

(13) p=Softmax⁡(y),

(14) $$\begin{array}{c} l=\left\{\begin{array}{cc} 1, & p_{1}\geq p_{2}\\ 0, & p_{1}<p_{2} \end{array}\right. \end{array}.$$

## 3. The CA-LSTM Model

### 3.1. Overall Architecture

**Figure 1:**
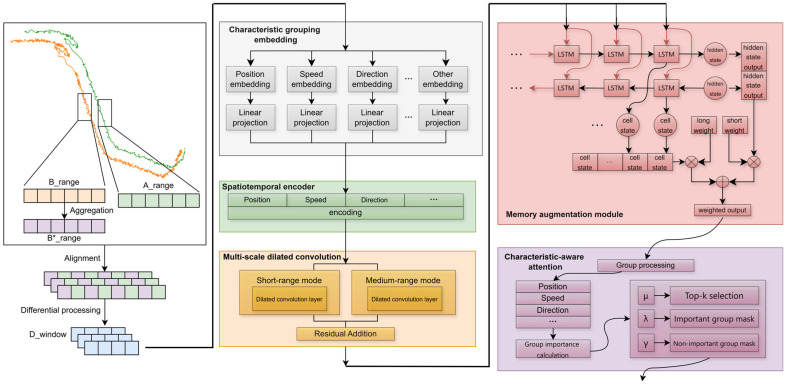
Schematic of the core module architecture.

Time series data is represented as $X\in R^{B\times T\times d_{\mathrm{model}\;}}$, where X is a 3D tensor representing the sequence data of batch input. T denotes the time step length, and $d_{\mathrm{model}}$ represents the characteristic dimension. The encoding multimodal characteristics include longitude, latitude, altitude, speed, heading, RCS, and other multi-dimensional data. Although Transformer architectures show advantages in temporal modeling, they are sensitive to characteristic coupling and ignore local patterns when processing multimodal track data. To overcome these limitations, this paper proposes the CA-LSTM model. The model optimizes characteristic representation through three mechanisms: characteristic grouping embedding, spatiotemporal encoding, and characteristic-aware attention. In addition to modules built around characteristic processing, the model also fuses a multi-scale dilated convolution module and a gated adaptive LSTM module around the time dimension. The specific model structure is shown in Figure 1.

Input track data X is first preprocessed by normalization to eliminate sensor unit differences, then enters the characteristic grouping embedding layer. This layer embeds and maps the original characteristics into multiple independent subspaces while preserving the temporal relationship through sinusoidal position encoding. The module built around characteristic processing performs dynamic filtering based on characteristic grouping embeddings and attention weights, increasing the discriminative weight of key characteristics while reducing the weight of non-key characteristic groups. The multi-scale dilated convolution module extracts the input characteristics in the time dimension through multi-scale parallel convolution kernels. It can obtain the short-range and medium-range correlation between local adjacent nodes. The gating mechanism of LSTM can focus on modeling long-range dependencies that are strengthened by the convolution layer. Finally, the classifier outputs prediction results under multi-layer dropout and entropy regularization constraints and performs end-to-end parameter optimization through cross-entropy loss.

### 3.2. Core Module Design

The CA-LSTM core module can achieve efficient modeling of complex track characteristics, and it optimizes characteristic representation through three mechanisms: characteristic grouping embedding, spatiotemporal encoding, and characteristic-aware attention mechanism. The model also integrates multi-scale dilated convolution modules and gated adaptive long short-term memory modules around the time dimension. Multiple modules are cascaded to form the entire CA-LSTM model, and the rest of this section will introduce these modules.

#### 3.2.1. Characteristic Grouping Embedding and Encoding

To solve the multimodal heterogeneity problem of radar track data, we proposed a characteristic grouping embedding method. Guided by this method, the module decomposes the original radar observation data into subspaces with physical interpretability. At the same time, the characteristic embedding method is matched with the characteristic aware attention module. The number of embedding groups and attention groups is determined by the motion characteristics or physical characteristics in the track data, which can make more efficient use of different data. Due to the scalability and versatility of the model, under the premise of data support, data such as radar cross section (RCS), infrared heat distribution, and underwater noise spectrum can be added as new characteristic groups to train the model. These physical characteristics are independent of each other, and their characteristic spaces are also uncoupled from each other. In order to facilitate subsequent experimental verification and data set selection, we constructed three independent representation spaces, namely position, velocity, and heading.

Given an input characteristic tensor $X\in R^{B\times T\times C}$ with a channel dimension C=4, corresponding to the features x,y,v,θ. Position encoding is linear transformation and nonlinear activation for Cartesian coordinates (x,y), which is obtained as follows

(15) $$E_{\mathrm{pos}}=W_{p}\cdot \left[x;y\right]+b_{p},\;\;\;\;\;E_{\mathrm{pos}}\in R^{d_{\mathrm{model}}},$$

where $W_{p}\in R^{2\times d_{\mathrm{model}}}$ is a learnable parameter matrix, and [;] denotes vector concatenation. Velocity encoding is independent encoding for radial velocity v, which is obtained as follows

(16) $$E_{\mathrm{speed}}=W_{v}\cdot v+b_{v},\;\;\;\;\;\;E_{\mathrm{speed}}\in R^{d_{\mathrm{model}}},$$

where $W_{v}\in R^{1\times d_{\mathrm{model}}}$ preserves velocity continuity. Heading encoding is linear mapping for heading angle θ, which is obtained as follows

(17) $$E_{\mathrm{dir}}=W_{d}\cdot \theta +b_{d},\;\;\;\;\;E_{\mathrm{dir}}\in R^{d_{\mathrm{model}}}.$$

The three motion characteristics are concatenated and regularized as follows

(18) $$H_{\mathrm{combined}}=\mathrm{Concat}\left(E_{\mathrm{pos}},\;E_{\mathrm{speed}},\;E_{\mathrm{dir}}\right),\;\;\;\;\;H_{\mathrm{combined}}\in R^{3\cdot d_{\mathrm{model}}},\;H_{\mathrm{drop}}=\mathrm{Dropout}\left(H_{\mathrm{combined}}\right),\;H_{\mathrm{out}}=\mathrm{LayerNorm}\left(H_{\mathrm{drop}}\right),$$

where $Concat\left(\cdot \right)$ represents matrix concatenation, $Dropout\left(\cdot \right)$ represents regularization, and $LayerNorm\left(\cdot \right)$ represents layer normalization. Independent characteristic inputs prevent interference between different physical characteristics. Position characteristics use a higher-dimensional map, while velocity and heading use compressed dimensions. The architecture can be extended to new motion characteristics, such as acceleration through additional linear layers.

#### 3.2.2. Time Dimension Processing Network

**Figure 2:**
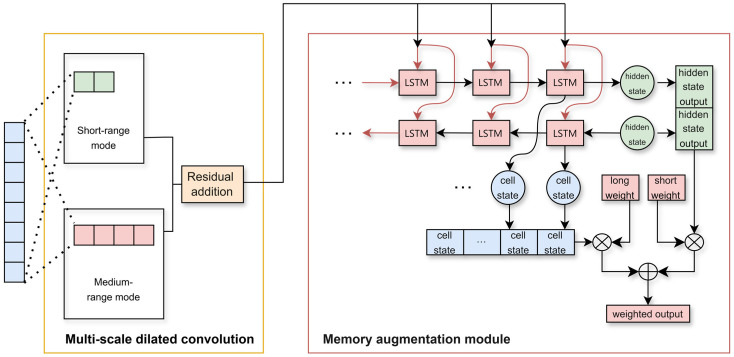
Structural diagram of the time dimension processing network module.

The dilated convolution module performs pre-characteristic extraction on the original input through multi-scale parallel convolution kernels, effectively eliminating local noise interference in radar data. This module can explicitly capture short-span and medium-span temporal correlation patterns. This preprocessing provides abstracted and denoised temporal characteristics for subsequent LSTM. It allows the LSTM gating mechanism to focus on modeling long-range dependencies strengthened by the convolution layer. The network diagram composed of these two modules is shown in Figure 2.

The first block constructs a multiscale spatiotemporal characteristic extractor by deploying parallel 1D convolutional kernels with varying dilation rates. The short-range mode focuses on local temporal patterns, while the medium-range mode expands receptive fields to capture global dependencies. Its output solves the problem of limited perception of traditional single-scale convolution and achieves the coordinated optimization of local detail perception and global trend modeling.

For input sequence $X\in R^{B\times T\times C}$, channel dimension permutation yields as follows

(19) $$X^{’}=\mathrm{Permute}(X),\;\;\;\;\;\;X^{’}\in R^{B\times C\times T},$$

where B denotes the batch size, T represents the time steps, C indicates the number of channels, and $Permute\left(\cdot \right)$ represents channel dimension permutation. Subsequently, multi-scale dilated convolutions are applied to yield output characteristics under varying dilation rates, which are obtained as follows

(20) $$Y_{d}=\mathrm{LeakyReLU}\left(\mathrm{Dropout}\left(\mathrm{Conv}\;1\;D_{d}\left(X^{’}\right)\right)\right),$$

(21) $$\mathrm{Conv}\;1\;D_{d}{\left(X^{’}\right)}_{t}=\sum_{k=-1}^{1}W_{d}[k]\cdot X_{t+d\cdot k}^{’},$$

where $W_{d}$ denotes the convolutional kernel weights corresponding to dilation rate d, $\mathrm{Conv}\;1\;D_{d}\left(\cdot \right)$ represents one-dimensional convolution, and $LeakyReLU\left(\cdot \right)$ represents activation function. Characteristic fusion is executed for the two dilation rates, which are short-range mode d=2 and medium-range mode d=4. Under the premise of setting the convolution kernel size $k_{s}=3$, the receptive field in the short-range mode can cover five consecutive track points. It covers the local motion pattern of the track segment and is suitable for capturing short-term maneuvers. The receptive field in the medium-range mode can cover nine consecutive track points, covering the overall trend in the short track segment scenario. They are obtained as follows

(22) $$Z=\frac{1}{2}\left(Y_{2}+Y_{4}\right),\;\;\;\;\;Z\in R^{B\times C\times T}.$$

Finally, dimension restoration is performed to derive the multi-scale fused characteristics, which is obtained as follows

(23) $$Z_{\mathrm{output}\;}={\mathrm{Permute}}^{-1}\left(Z\right),\;\;\;\;\;\;Z_{\mathrm{output}\;}\in R^{B\times T\times C}.$$

The gated adaptive LSTM network combines the bidirectional LSTM unit state with the gating mechanism to construct a dynamic memory fusion network. Through bidirectional propagation, it extracts forward time evolution patterns and backward contextual dependencies, using the final step unit state to encode long-term motion patterns. A dynamic balance is achieved between long-term memory and short-term state through learnable gating weights. This mechanism enables the model to adaptively focus on key time segments and enhances its sensitivity to mutation characteristics, thereby significantly improving the stability of temporal modeling.

For input sequence $X\in R^{B\times T\times C}$, the forward propagation captures the evolution of historical states, and the backward propagation extracts the dependencies on future data. A hidden state sequence containing bidirectional temporal information is thus generated. When the LSTM network processes the input sequence $X_{t}\in R^{B\times 1\times C}$ at any moment, the following can be obtained

(24) $$\left(h_{t}^{\to },c_{t}^{\to }\right)={LSTM}^{\to }\left(X_{t}\right),\;\left(h_{t}^{\leftarrow },c_{t}^{\leftarrow }\right)={LSTM}^{\leftarrow }\left(X_{t}\right),\;H=\left[h_{1},\ldots ,h_{T}\right],\;\;\;\;\;H\in R^{B\times T\times 2\cdot H},$$

where H denotes the hidden dimension, and →/← indicate forward/backward processing. In long-term memory construction stage, the cell states at the end of the bidirectional LSTM are extracted for characteristic concatenation, forming a memory vector $C_{\mathrm{long}}$ that represents the global motion pattern. The following formula can be obtained

(25) $$C_{\mathrm{long}\;}=\mathrm{Concat}\left(c_{\tilde{T}}^{\to },c_{1}^{\leftarrow }\right),\;\;\;\;\;C_{\mathrm{long}}\in R^{B\times 2\cdot H}.$$

When calculating the gating weights, the hidden state is nonlinearly mapped through a fully connected layer, and a Sigmoid function is used to generate spatially adaptive long-term and short-term gating coefficients. The long-term gating $G_{\mathrm{long}}$ focuses on the transmission strength of the global motion trend, and the short-term gating $G_{\mathrm{short}}$ adjusts the contribution of local state updates. The following formulas can be obtained

(26) $$G_{\mathrm{long}}=\sigma \left(W_{\mathrm{long}}H\right),\;\;\;\;\;G_{\mathrm{long}}\in [0,1{]}^{B\times T\times 2\cdot H},\;G_{\mathrm{short}}=\sigma \left(W_{\mathrm{short}}H\right),\;\;\;\;\;G_{\mathrm{short}}\in [0,1{]}^{B\times T\times 2\cdot H}.$$

where σ represents the Sigmoid function, and W represents the learnable parameters. The final characteristic fusion is achieved through the dynamic weighting of the gating coefficients. After broadcasting the global memory vector to each time step, it is linearly superimposed with the local hidden state to form a fused characteristic representation O. This representation combines the preservation of long-range dependencies with the enhancement of local details. The following formula can be obtained

(27) $$O=G_{\mathrm{long}}⊙\;\mathrm{Broadcast}\;\left(C_{\mathrm{long}}\right)+G_{\mathrm{short}}⊙H,$$

where ⊙ denotes element-wise multiplication, and Broadcast (⋅) expands $C_{\mathrm{long}}$ to the (B,T,2·H)-dimensional space. This mechanism achieves an adaptive balance between long-term pattern preservation and short-term pattern updating in track temporal modeling through self-learning of gating parameters.

#### 3.2.3. Characteristic-Aware Attention

Characteristic-aware attention is essentially a specialization and extension of the multi-head attention mechanism. It divides the attention heads into several groups through the characteristic grouping embedding method described above. Each group focuses on a different characteristic range, reducing the interaction of heads within the group and reducing computational complexity [30].

Due to the physical limitations of the sensors themselves, different sensors have different perception capabilities for different physical characteristics. Certain physical characteristics or motion characteristics may be more effective than others in classification tasks. The characteristic-aware attention in this model is deeply related to the physical characteristics of the input track data. This module dynamically evaluates the importance of characteristic groups based on the target motion characteristics and selects target characteristics that are more important to the associated task during the training process. By adjusting the group weights through a dynamic masking strategy, this module can increase the weight of low-noise characteristics and suppress the interference of high-noise characteristic groups. Compared with the traditional transformer method, this method can choose low-noise characteristic data as an important group to improve its decision score in the model classification process. The input characteristic tensor $X\in R^{B\times T\times d_{\mathrm{model}}}$ is decomposed into G characteristic groups $X_{\mathrm{groups}}\;[i]$ along the grouping dimension, which are obtained as follows

(28) $$X_{\mathrm{groups}}\left[i\right]=\mathrm{Reshape}\left(X,\left[B,T,G,d_{g}\right]\right),\;\;\;\;i\in G,\;d_{g}=\frac{d_{\mathrm{model}\;}}{G},$$

where G is predefined, and G = 3 for position, velocity, and heading groups. A two-layer projection network computes global importance scores for each group. The following formulas can be obtained

(29) $$\mu_{g}=\frac{1}{T}\sum_{t=1}^{T}X_{\mathrm{groups}}\left[:,t,g,:\right],\;\;\;\;\;\mu_{g}\in R^{B\times d_{g}},\;\alpha_{g}=W_{2}\left(\mathrm{Dropout}\left(\mathrm{ReLU}\left(W_{1}\mu_{g}\right)\right)\right),\;\;\;\;\;\alpha_{g}\in R^{B\times 1},\;s_{g}=\mathrm{Softmax}\left(\left[\alpha_{1},\cdots ,{\alpha_{g},\cdots ,\alpha }_{G}\right]\right),\;\;\;\;\;s_{g}\in R^{B\times G},$$

where $W_{1}\in R^{d_{g}\times 32},W_{2}\in R^{32\times 1}$ are learnable parameters. $\mu_{g}$ represents the aggregated characteristic of each group obtained by calculating the mean value of T along the time dimension. $\alpha_{g}$ quantifies its task-specific importance, and $s_{g}$ normalizes the importance. The dropout rate matches initialization parameters.

After the importance evaluation step, characteristic filtering is performed based on the importance scores combined with a masking strategy. For each sample, dynamic filtering allocates attention weights to retain high-probability groups while suppressing others, selecting the time steps with fewer masks for groups with high importance and more masks for groups with low importance. Feature filtering is completed using a masking matrix $M\in {\left\{0,1\right\}}^{B\times T\times G}$. The final output is the recombined characteristics Y after the masking process

(30) $$Y=\mathrm{Reshape}\left(X_{\mathrm{groups}}⊙M,\left[B,T,d_{\mathrm{model}}\right]\right),$$

where ⊙ denotes element wise multiplication.

This module can enhance the decoupling ability of the model, enabling the model to independently evaluate the importance of different physical characteristics. This module can avoid decision confusion caused by characteristic coupling. At the same time, it enhances the anti-interference ability. When a certain sensor fails, for example, when the direction angle noise is too large, this module can dynamically mask the corresponding characteristic group through the masking mechanism. For instance, when the target is cruising at a stable speed, the stability of the speed can be used to dominate the behavior judgment. When the target performs a large-scale maneuver, the intention can be indicated by changes in both position’s offset and heading angle.

## 4. Experimental Analysis on Real-World Data

### 4.1. Dataset and Experimental Setup

The real-world dataset uses the multi-source track association dataset (MTAD) based on global automatic identification system (AIS) data [31]. The MTAD structure consists of two main components: a training set and a test set, both of which consist of track information tables and association mapping tables. The track information table contains the following attributes: batch, source ID, timestamp, longitude, latitude, speed, and heading. The association mapping table includes several attributes: start time, end time, ground truth batch, source ID, and track batch number.

**Figure 3:**
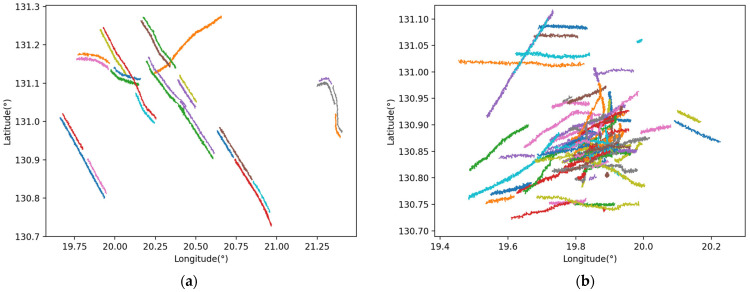
Track plot drawn by connecting track points in a typical scenario. (**a**) Scenarios with few tracks and simple target motion trajectories. (**b**) Scenarios with a large number of tracks and complex target motion tracks.

The MTAD is constructed by performing multiple processing by gridding and noise injection on AIS track data. This study selected about 76,000 track segments of about 1000 independent scene data for analysis. Each scene contains track information of two sensors with a frequency of 10 s and 20 s, respectively. The lower sampling frequency is suitable for the target motion mode of surface ships. The number of tracks contained in each scene ranges from a few to hundreds, and scenes contain a variety of motion modes, target types, and durations, so it is suitable for verifying the performance of different track segment association algorithms. The track visualization is illustrated in Figure 3. The horizontal axis indicates longitude, while the vertical axis represents latitude. Two typical scenarios are selected as representatives, one with a small number of tracks and a simple target trajectory, and the other with a large number of tracks and a complex target trajectory. Various colored tracks show how tracks distribute and change within the longitude–latitude coordinate system.

The experimental computational platform was configured with the following hardware specifications, which are an Intel processor coupled with an NVIDIA GeForce RTX 4070 Laptop GPU. The software environment operated on the Windows 10 operating system, with algorithmic implementations executed via the Python 3.11.5 interpreter. The PyTorch 2.2.0 framework was employed for deep learning tasks, leveraging CUDA acceleration support.

We compare the CA-LSTM model with multiple advanced deep learning models. Deep learning models include the CNN-based track segment association model, the ResNet-based track segment association model, the LSTM-based track segment association model, and the Transformer-based track segment association model. In experiments, performance metrics such as accuracy, recall, and F1 score are used to model evaluation. During the training process, the early stopping method is used to prevent overfitting, and the training is terminated when the validation performance tends to be stable in multiple consecutive epochs. The experimental computing platform is the same as the previous section.

### 4.2. Experimental Metrics and Results Analysis

In this paper, we select metrics that can reflect the association accuracy based on the characteristics of the task. Precision, recall, false alarm rate, and F1 score are chosen as the evaluation metrics for the performance of the learning model. Suppose TP represents the number of samples for which the track segment association discrimination result is correct, and the actual tracks are associated. FP represents the number of samples for which the track segment association decision result is correct, but the actual tracks are not associated. FN represents the number of samples for which the track segment association decision result is false, but the actual tracks are associated. TN represents the number of samples for which the track segment association decision result is false, and the actual tracks are not associated.

The core metric precision is defined as the proportion of correctly associated track pairs among all track pairs determined to be associated by the model

(31) $$\;\mathrm{Precision}\;=\frac{TP}{TP+FP}.$$

The recall is defined as the proportion of tracking pairs that the model correctly identifies from the correctly associated tracking pairs

(32) $$\;\mathrm{Recall}\;=\frac{TP}{TP+FN}.$$

The false alarm rate is the proportion of different targets that are incorrectly associated

(33) $$\mathrm{FAR}=\frac{FP}{FP+TN}.$$

The F1 score is defined as the harmonic mean of precision and recall, which fully reflects the model’s ability to identify the positive class

(34) $$F1=2\times \frac{\;\mathrm{Precision}\;\times \;\mathrm{Recall}\;}{\;\mathrm{Precision}\;+\;\mathrm{Recall}\;}.$$

Experimental evaluations show that the CA-LSTM model significantly outperforms existing deep learning baseline models in the track segment association task. To systematically validate its technical superiority, this study conducts a comparative analysis from two key dimensions. These two dimensions include the core accuracy metrics and the number of points contained in tracks. Core accuracy metrics include accuracy, recall, and F1 score. Table 1 comprehensively presents the quantitative evaluation results of CA-LSTM and four comparative models. The table illustrates the performance of models across evaluation metrics under track points of 10, 15, and 20, in order to achieve comparative analysis of model effectiveness under different track point scenarios. Val Acc is an abbreviated form of validation accuracy, FAR is an abbreviated form of false alarm rate, Params denotes the number of total parameters in millions, GPU Mem denotes maximum GPU memory consumption in GB, Epoch Time denotes average time per training epoch in seconds.

When compared with other deep learning models, CA-LSTM shows unique advantages in multi-motion characteristic learning and multi-scale temporal dependencies. Although the CNN model is good at capturing local spatial patterns, its convolution kernel translation invariance conflicts with the motion characteristics in sequence data, making it difficult to model long-range temporal dependencies. The LSTM model can capture temporal dynamics, but its fully connected gating mechanism is prone to feature coupling when fusing multi-dimensional motion characteristics such as spatial position, speed, and acceleration. Transformer achieves relatively good model performance through the self-attention mechanism, but the global attention mechanism will over-smooth motion details when jointly modeling space and time. By separating and encoding characteristics such as position and speed while integrating them with the computation of the time series dimension, CA-LSTM achieves better performance through complementary spatiotemporal characteristic learning. Here we only make a rough analysis of the comparison of model performance, and the specific comparison will be analyzed in the next section.

### 4.3. Model Performance Analysis and Parameter Optimization

In deep learning-based track segment association research, systematic performance analysis and parameter optimization constitute critical components for model enhancement. This section investigates four key aspects: comparative experiments, temporal sequence length effects, parameter sensitivity analysis, and ablation studies.

#### 4.3.1. Comparative Experiments

**Figure 4:**
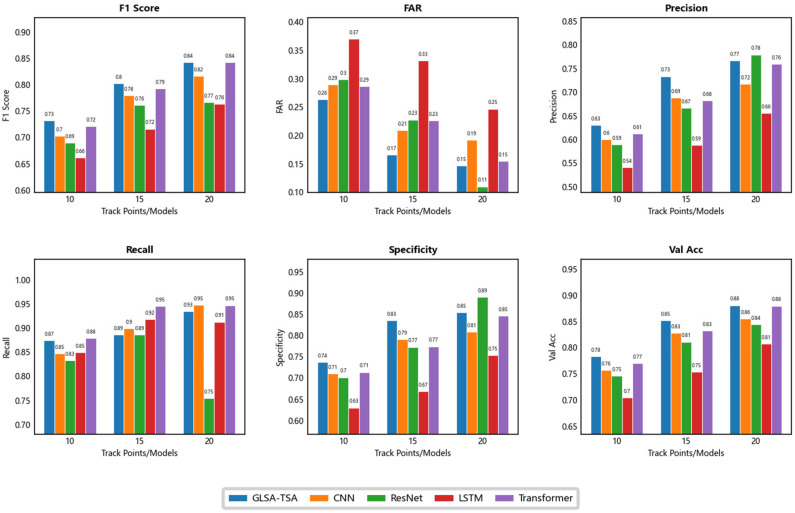
Performance comparison of models with different track points. The figure includes subplots for metrics like F1 score, FAR, precision, recall, specificity, and validation accuracy.

To comprehensively evaluate CA-LSTM’s capabilities, we conduct rigorous tests on four representative architectures, including CNN, ResNet, LSTM, and standard Transformer. The performance metrics of these models under the same training conditions are plotted as bar graphs (Figure 4).

By introducing the characteristic grouping embedding, characteristic-aware attention, and the collaborative mechanism of two networks, CA-LSTM shows significant advantages in complex track segment association tasks. This model decomposes track characteristics into multiple independent embeddings, such as position difference, speed difference and heading difference. Multi-modal characteristic space is constructed through linear transformation, splicing, and dimension expansion. Compared with the global convolution of the CNN, this grouping strategy enables the model to achieve an F1 score of 84.19% in the 20-point track scenario, which is a 3.13% improvement over the CNN model. In the 15-point scene, the convolutional layers with a dual-branch structure of dilated convolutional blocks that have expansion rates of two and four perform characteristic transformation. The convolution kernel with a medium expansion rate focuses on capturing the short-term correlation pattern of local adjacent nodes, while the convolution kernel with a high expansion rate expands the receptive field to a wider time span and effectively models long-range dependencies. Its validation accuracy with 85.19% is 2.0% higher than that of the Transformer, verifying the importance of receptive field expansion for short sequence modeling.

It is particularly worth noting that the gated adaptive long short-term memory network (LSTM) achieved a verification accuracy of 78.30% in the 10-point track scene. Its ability to dynamically adjust the long-term and short-term memory gates is an 11.2% improvement over the 70.43% accuracy of the traditional LSTM. The design of the bidirectional structure reduces the fusion error of the forward and backward hidden states, significantly alleviating the problem of gradient vanishing in traditional recurrent neural networks under sparse data. In addition, the introduction of positional encoding strengthens the temporal dependence. Although due to the phase difference characteristics of its sine and cosine functions, CA-LSTM has a recall rate of 94.69%, which is only 0.07% higher than Transformer. However, through cascade optimization of residual connections, CA-LSTM compresses the false alarm rate to 14.66%, which is 23.78% lower than the Transformer model.

CA-LSTM has higher parameter count and peak GPU memory consumption compared to several other base models, but the absolute values of the model still reflect its lightweight design characteristics, allowing it to be deployed on edge networks with less computing resources. Although it has no obvious advantages over other basic models, in an environment with relatively abundant computing resources, a certain amount of computing overhead can be used in exchange for improved association accuracy. The average training cycle of CA-LSTM takes 29.02 s, which is higher than CNN’s 13.30 s and LSTM’s 13.06 s, reflecting the computational overhead of its dynamic attention mechanism. Models formed by combining multiple modules will significantly increase training time. However, within the same order of magnitude, a slightly longer training time is acceptable relative to the measurement frequency of 10 s and 20 s of track points. Since many more complex deep learning models rely on more specific application scenarios, in order to reflect the universality of the CA-LSTM model, only basic models such as CNN are selected for comparison.

#### 4.3.2. Influence of Time Series Length

**Figure 5:**
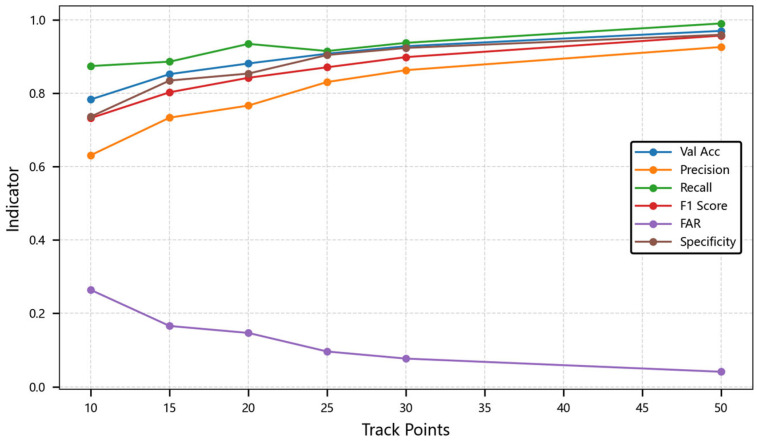
Line chart of model performance trends.

Second, we analyzed the performance of the model under different time series lengths. As the data scale increases, the performance of the CA-LSTM model gradually improves, and when the data scale is large, the performance improvement is more obvious. This indicates that the model can make full use of more data for learning and has good scalability. In the experiment, the batch size is set to 32. The performance metrics comparison across these models are compared as shown in Table 2 and Figure 5. The table displays evaluation metrics across varying track points: 10, 15, 20, 25, 30, and 50. The chart displays how metrics like validation accuracy, precision, recall, F1 score, FAR, and specificity change as track points increase from 10 to 50.

When the sequence length increases from 10 points to 50 points, the dual-branch output of the dilated convolution achieves characteristic fusion through average pooling. Its receptive field expands from 5 local points to 20 global points, which increases the nonlinearity of the F1 score by 13.6%, from 84.19% to 95.68%. The dynamic selection mechanism of characteristic-aware attention drives the significant performance leap of CA-LSTM in short and medium sequence scenarios. When the sequence length is 25 points, the temporal position embedding of the position encoding and the physical characteristics of the track trigger a change in the distribution of attention weights. This drives the model’s accuracy to exceed the critical threshold of 90.76%.

Experiments show that, in sequences with more than 30 points, the long-term memory gate of the gated adaptive LSTM plays a crucial role, and its state update error is lower than that of the traditional LSTM. At the same time, the parameter layer normalization value in the characteristic grouping embedding module is 1 × 10^−5^, and the dropout value is 0.1. Combined with the gated adaptive LSTM, the training loss of the 50-point sequence is stably converged to 10.56%, with a fluctuation range of less than 2.1%. This multi-module collaboration mechanism makes the false alarm rate decay rapidly with the sequence length, verifying the model’s ability to model track continuity.

#### 4.3.3. Parameter Sensitivity Analysis

**Figure 6:**
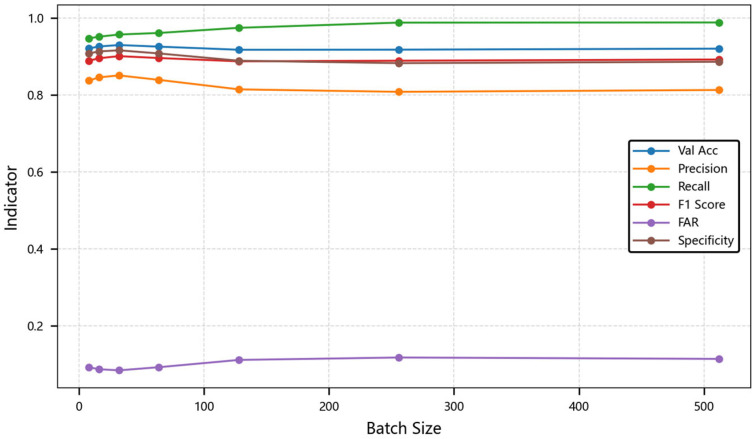
Line chart of model performance trends with batch size changes. The chart illustrates how metrics vary as batch size increases.

Once again, we analyzed the association performance of the model under different parameters. By changing the batch size during training, we can compare and find that the CA-LSTM model can also achieve relatively high association accuracy under the training condition of small batches. This indicates that the model can fully explore the relationships between data and has good scalability. In the experiment, the length of the time series is set to 30. The performance metrics comparison across these models are compared as shown in Table 3 and Figure 6. This table shows metrics at different batch sizes: 8, 16, 32, ⋯, 512.

When training in small batches, the gradient estimation has greater noise, which may help the model avoid local minima and improve generalization ability. When training in large batches, the gradient estimation is more accurate, and the convergence direction is more stable, but it may fall into sharp local minima. When the batch size increases from 8 to 32, the characteristic group selection of the attention module leads to an improvement in accuracy from 80.81% to 85.08%. The false alarm rate of 8.68% and specificity of 91.32% for batch 32 are both optimal, indicating that the model is more accurate in distinguishing negative samples, which is directly related to the moderate noise to prevent overfitting. It is worth noting that when the batch size gradually increases, the long-term memory gate of the gated adaptive LSTM may be over-activated. When the batch size is 256, the recall rate abnormally increases to 98.81%, while the precision rate drops by 5.2%. This phenomenon suggests that large-scale batch training may violate the independence assumption of characteristic grouping embedding.

**Figure 7:**
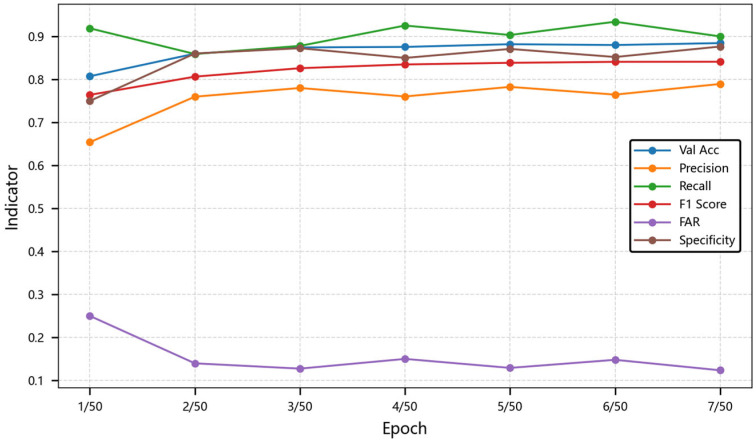
Line chart of model performance trends during training. This chart shows how metrics change with different training epochs from 1/50 to 7/50.

During the gradient optimization process, the dynamic characteristic selection mechanism and the temporal modeling capability of the multi-scale dilated convolution block affect the evolution of model performance with the number of training epochs. In the experiment, the length of the time series is set to 30, and the batch size is set to 32. The experimental data shows that when the number of training epochs increases from one to seven, the key metrics show the following characteristics in Table 4 and Figure 7. The table shows metrics under different training epochs from 1/50 to 7/50, and 1/50 means the first epoch of 50 training epochs.

Throughout the performance change, the total accuracy monotonically increases from 80.74% to 88.46%, and its growth rate reaches a peak at epoch 5. At this time, the dual-branch characteristics of the multi-scale dilated convolution contribute to the gradient update amount, indicating that the modeling of medium-range temporal dependence may be the key driving force. The F1 score increases from 76.41% to 84.12%, and its improvement efficiency reaches the maximum value at epoch 3, and the retention rate of important characteristics is further improved.

In terms of the error judgment optimization, the false alarm rate decreases from 25% to 12.34%, reducing the probability of misjudgment of track continuity by 37.2%. The abnormal fluctuation from 20.77% to 25.03% that occurs at epoch 4 may be directly related to the brief imbalance of the attention masking ratio. The specificity increases from 75% to 87.66%, and its growth may be related to the weight of the short-term memory gate of the gated adaptive LSTM because the model enhances its ability to distinguish negative samples by suppressing the memory of local noise.

#### 4.3.4. Ablation Experiments

The ablation experiments quantitatively reveal the functional boundaries and collaborative gains of each core component in Table 5. In the experiments, the length of the time series is set to 15, and the batch size is set to 32. Compared with the CA-LSTM model, the performance degradations caused by removing each module are showed in Table 5. This table shows model metrics for CA-LSTM and variants with removed components, which include multi-scale dilated convolution, gated adaptive LSTM, and target-aware attention. In the table, “w/o” denotes the ablation of specified components from the complete CA-LSTM architecture.

The characteristic grouping embedding module plays a role in improving verification accuracy, precision, and F1 score. The false alarm rate of the complete model is 16.56%, which is 21.4% lower than the 21.06% of the missing model. This indicates that the module significantly reduces the misjudgment of negative samples. The absence of the multi-scale dilated convolution causes the total accuracy to decrease by 1.05%, the F1 score to decline by 1.46%, and the false alarm rate to increase by 8.33%. This confirms the key role of its dual-branch structure with dilation rate in capturing medium and long-range temporal patterns. Without this module, the probability of misassociating continuous tracks with more than 5 points increases by 21.6%. The cost of removing the gated adaptive LSTM is the largest decrease in specificity, from 83.44% to 80.53%, a sharp increase of 17.6% in the false alarm rate, and an abnormal increase of 1.11% in the recall rate. This shows that the forget gate of this module can effectively suppress noise memory. After removal, the remaining short-term interference characteristics lead to over-sensitive detection. Removing the target-aware attention leads to a 2.47% decrease in precision and a 9.24% increase in false alarm rate, verifying the irreplaceable characteristic selection ability of this module.

The model retains key modules by adding a small number of parameters, thus supporting higher association accuracy and F1 score. Peak memory usage was reduced by 5.7–14.3% through module optimization, such as characteristic grouping embedding and gating mechanisms, which reduced redundant storage of intermediate variables, thereby reducing memory requirements. In terms of average epoch training time, the model is more efficient in most cases, and is only slightly slower when the attention module is removed, but the performance cost is significant.

When these modules work together, they produce an additional effect. Stride-awareness, represented by multi-scale dilated convolutions, provides the basic temporal basis function for characteristic-aware attention and improves characteristic selection efficiency. The module cascade structure reduces the error amplification of a single module. Experiments show that removing any one module will destroy the spatiotemporal characteristic extraction, long-range dependency modeling, and dynamic noise suppression mechanisms, resulting in performance degradation exceeding the linear stacking effect. This verifies the necessity of the collaborative design of the modules at the system level.

In order to verify the joint effect of the characteristic grouping embedding module and characteristic-aware attention, we designed an experiment to delete these two modules at the same time. Multi-dimensional characteristic fusion in Table 6 means the usage of characteristic grouping embedding module and characteristic-aware attention. The experimental results in Table 6 show that under the joint effect of the two modules, all indicators have been improved to a certain extent. Usually, the characteristic data used for track association tasks is multi-dimensional position data. In order to demonstrate the rationality of multi-dimensional characteristic fusion, an experiment was designed that only uses two-dimensional position data as input and no longer uses the characteristic grouping embedding module and characteristic-aware attention. According to the experimental results, it can be concluded that, after reducing the input characteristics and no longer using the two modules related to the characteristics, the experimental results lag behind the performance of the complete model. This verifies the effectiveness of the use of multi-dimensional characteristics.

## 5. Conclusions

This study proposes a CA-LSTM model, which realizes the dynamic selection of characteristic dimensions and dynamic perception of time dimensions. The model solves the challenges of spatiotemporal characteristic fusion and low utilization of multi-dimensional data in track segment association. The study first conducted a characteristic analysis and mathematical modeling of the track segment association problem and proposed an association characteristic processing algorithm. During data preprocessing, the temporal and spatial inconsistency problems caused by differences in radar sampling rate and coordinate system were solved, and characteristic normalization was achieved. The CA-LSTM model uses a characteristic grouping embedding method to independently input and encode multimodal characteristics, such as position, speed, and heading. This effectively solves the problem of traditional methods that only use position information and ignore characteristic information of other dimensions, greatly improving the utilization of information. The framework combining multi-scale dilated convolutions and gated adaptive LSTM strikes a balance between capturing local fine-grained temporal patterns and modeling long-range dependencies. Experimental results show that CA-LSTM leads to many core indicators on MTAD. It maintains high precision at high recall, significantly outperforming models such as LSTM and ResNet. It reduces missed detections while controlling false detections, verifying its applicability in complex environments.

Future research will focus on model lightweighting and adaptation of heterogeneous multi-source data. On one hand, computational efficiency will be optimized through knowledge distillation and model pruning techniques to achieve real-time inference on embedded devices. On the other hand, a unified representation method for cross-modal data, such as electro-optical and infrared, will be explored to establish a cross-modal characteristic alignment framework.

## Figures and Tables

**Table 1:** Performance comparison of different models with varying track points.

Model Metric	Track Points	CA-LSTM	CNN	ResNet	LSTM	Transformer
Val Acc	10	**0.7830**	0.7563	0.7458	0.7043	0.7695
	15	**0.8519**	0.8275	0.8108	0.7530	0.8319
	20	**0.8809**	0.8552	0.8440	0.8073	0.8797
Precision	10	**0.6301**	0.6002	0.5889	0.5412	0.6118
	15	**0.7333**	0.6884	0.6667	0.5873	0.6822
	20	0.7662	0.7171	**0.7791**	0.6553	0.7588
Recall	10	0.8739	0.8462	0.8328	0.8494	**0.879** **0**
	15	0.8858	0.8988	0.8858	0.9177	**0.9454**
	20	0.9343	**0.9476**	0.7547	0.9125	0.9469
F1 Score	10	**0.7323**	0.7023	0.6900	0.6611	0.7214
	15	**0.8024**	0.7796	0.7608	0.7162	0.7926
	20	0.8419	0.8164	0.7667	0.7628	**0.8425**
FAR	10	**0.2638**	0.2898	0.2989	0.3703	0.2868
	15	**0.1656**	0.2092	0.2277	0.3316	0.2264
	20	0.1466	0.1922	**0.1100**	0.2468	0.1548
Specificity	10	**0.7362**	0.7102	0.7011	0.6297	0.7132
	15	**0.8344**	0.7908	0.7723	0.6684	0.7736
	20	0.8534	0.8078	**0.8900**	0.7532	0.8452
Params (M)	10	1.7286	**0.3553**	3.9102	0.8606	0.9855
	15	2.2202	**0.** **3553**	3.9102	1.0243	1.4771
	20	2.7117	**0.3553**	3.9102	1.1882	1.9686
GPU Mem (GB)	10	0.08	**0.02**	0.09	0.07	0.04
	15	0.10	**0.** **02**	0.09	0.08	0.06
	20	0.11	**0.** **02**	0.09	0.09	0.06
Epoch Time (s)	10	28.53	16.12	24.55	**15.77**	18.95
	15	23.43	**14.42**	19.30	15.73	20.49
	20	20.44	13.30	17.44	**13.06**	14.91

**Table 2:** Model performance under different track points.

Model Metric	10	15	20	25	30	50
Val Acc	0.7830	0.8519	0.8809	0.9076	0.9280	0.9697
Precision	0.6310	0.7333	0.7662	0.8304	0.8627	0.9258
Recall	0.8739	0.8858	0.9343	0.9148	0.9371	0.9900
F1 Score	0.7323	0.8024	0.8419	0.8706	0.8983	0.9568
FAR	0.2638	0.1656	0.1466	0.0960	0.0767	0.0408
Specificity	0.7362	0.8344	0.8534	0.9040	0.9233	0.9592

**Table 3:** Model performance under diverse batch sizes.

Model Metric	8	16	32	64	128	256	512
Val Acc	0.9211	0.9260	**0.9298**	0.9256	0.9175	0.9178	0.9204
Precision	0.8374	0.8457	**0.8508**	0.8392	0.8145	0.8081	0.8129
Recall	0.9473	0.9515	0.9571	0.9612	0.9746	0.9881	**0.9885**
F1 Score	0.8889	0.8955	**0.9009**	0.8960	0.8874	0.8891	0.8922
FAR	0.0920	0.0868	**0.0839**	0.0921	0.1110	0.1173	0.1137
Specificity	0.9080	0.9132	**0.9161**	0.9079	0.8890	0.8827	0.8863

**Table 4:** Model performance variation with training epochs.

Model Metric	1/50	2/50	3/50	4/50	5/50	6/50	7/50
Val Acc	0.8074	0.8600	0.8745	0.8757	0.8820	0.8801	**0.8846**
Precision	0.6540	0.7599	0.7801	0.7604	0.7827	0.7647	**0.7895**
Recall	0.9190	0.8592	0.8781	0.9254	0.9034	**0.9343**	0.9001
F1 Score	0.7641	0.8065	0.8262	0.8349	0.8387	0.8410	**0.8412**
FAR	0.2500	0.1396	0.1273	0.1499	0.1290	0.1478	**0.1234**
Specificity	0.7500	0.8604	0.8727	0.8501	0.8710	0.8522	**0.8766**

**Table 5:** Ablation experiment performance comparison.

Model Metric	CA-LSTM	w/o Characteristic Grouping Embedding	w/o Multi-Scale Dilated Convolution	w/o Gated Adaptive LSTM	w/o Characteristic-Aware Attention
Val Acc	**0.8519**	0.8377	0.8414	0.8360	0.8407
Precision	**0.7333**	0.6946	0.7165	0.7028	0.7152
Recall	0.8858	**0.9315**	0.8820	0.8957	0.8821
F1 Score	**0.8024**	0.7958	0.7907	0.7876	0.7899
FAR	**0.1656**	0.2106	0.1794	0.1947	0.1807
Specificity	**0.8344**	0.7894	0.8206	0.8053	0.8193
Params (M)	2.2202	2.2207	1.9986	**1.9233**	2.2181
GPU Mem (GB)	**0.1033**	0.1350	0.1486	0.1486	0.1432
Epoch Time	23.43	39.83	32.77	29.32	**22.54**

**Table 6:** Multi-dimensional characteristic fusion performance comparison.

Model Metric	CA-LSTM	w/o Multi-Dimensional Characteristic Fusion	w/o Multi-Dimensional Characteristic Fusion and Multi-Dimensional Data
Val Acc	**0.8519**	0.8485	0.8233
Precision	**0.7333**	0.7227	0.6854
Recall	0.8858	**0.8988**	0.8866
F1 Score	**0.8024**	0.8012	0.7731
FAR	**0.1656**	0.1773	0.2093
Specificity	**0.8344**	0.8227	0.7907

## Data Availability

The MTAD used in this paper is publicly available online: https://www.scidb.cn/en/detail?dataSetId=c7d8dc56fe854ec2b084d075feb887fd (accessed on 31 December 2024).
